# Mitochondria-targeted antioxidant SkQ1 inhibits age-dependent involution of the thymus in normal and senescence-prone rats

**DOI:** 10.18632/aging.100043

**Published:** 2009-04-22

**Authors:** Lidia A. Obukhova, Vladimir P. Skulachev, Natalia G. Kolosova

**Affiliations:** ^1^Institute of Cytology and Genetics, Siberian Division of the Russian Academy of Sciences, Novosibirsk 630090, Russia; ^2^A.N. Belozersky Institute of Physico-Chemical Biology, Moscow State University, Moscow 119991, Russia; ^3^Novosibirsk State University, Novosibirsk 639090, Russia

**Keywords:** aging, thymus, progeric OXYS rats, mitochondrial-targeted antioxidant SkQ1

## Abstract

One of the most striking changes during mammal aging is a progressive involution of the thymus, associated with an increase in susceptibility to infections, autoimmune diseases and cancer. In order to delay age-related processes, we have developed mitochondria-targeted antioxidant plastoquinonyl decyltriphenyl phosphonium (SkQ1). Here we report that, at low doses, SkQ1 (250 nmol/kg per day) inhibited age-dependent involution of the thymus in normal (Wistar) and senescence-prone (OXYS) rats. SkQ1 preserved total weight and volume of the organ, the volume of thymic cortex and medulla, the thymic cellularity, and the number of CD3+, CD4+, and CD8+ cells in the thymus. Moreover, SkQ1 was especially effective in senescence-prone rats.   Thus SkQ1 slows down age-linked decline of the immune system, explaining prevention by this compound of infection-caused death in rodents, previously described in our group.

## Introduction

Aging is characterized by progressivedecline in the efficiency of physiological functions, resulting in an increased vulnerability of organisms to environmental stressors and in a growing risk of disease and death [1]. One of the most striking changes that occur during normal human aging is immunosenescence, a pro-gressive and generalized deterioration of immune functions that affects all cells and organs of the innate and adaptive immune systems. Progressive involution of the thymus, a hallmark of aging, leads to a decline of those immune functions that are related to T cell-depen- dent immunity and are generally associated with an increase in susceptibility to infections as well as increased incidence of autoimmune phenomena and cancer in the elderly [2,3]. The mechanism that underlines involution of the thymus is not well understood. Elucidation of such mechanisms is required to find a rational therapy that can prevent (or even reverse) lowering immunity in aged individuals [4,5]. Such a strategy will be in line with manifesto of Blagosklonny, Campisi and Sinclair published in the first issue of this journal [6].

There are several potential therapeutic approaches now available to slow immunosenescence. Among them is antioxidant treatment, which has been shown to be beneficial for immune cell function in the elderly [7,8]. Since Harman (1956) [9], who formulated the free radical theory of aging, antioxidants have been studied as anti-aging drugs in many laboratories. Fast senescence-prone animals suffering from oxidative stress are often a subject of such studies. One of these models, namely OXYS rats, was elaborated in our group. OXYS rats show significantly shortened lifespan (maximal lifespan is 28% shorter than that of Wistar rats) and early development of age-associated patho-logical phenotypes similar to several age-linked disorders observed in humans, including cataract, retinopathies, senile osteoporosis, high blood pressure, and higher levels of oxidative damage to lipids and proteins. Behavior of rather young OXYS rats is similar to the behavior of old Wistar rats and can be improved by treatment with antioxidants [10-14]. A premature invo-lution of the thymus and a decline of function of T cell-dependent immunity is inherent in OXYS rats [15,16].

Mitochondrial dysfunctions increasing with age are thought to be a causal factor for accelerated senescence in OXYS rats [16-19]. If this were the case, antioxidants might delay senescence in OXYS rats. Some indications supporting this has already been published [12,20]. Recently, we showed that nanomolar amounts of mitochondria-targeted antioxidant SkQ1 (plastoquino-nyl decyltriphenyl phosphonium) are capable of preventing some consequences of accelerated sense-cence in OXYS rats. One of the important advantages of SkQ1 is its rapid reduction by the mitochondrial respiratory chain complex III, i.e. SkQ1 is a rechargeable antioxidant [21]. According to our data, addition of very small amounts of SkQ1 to food completely prevented development of cataract and retinopathies in OXYS rats up to the age of two years [13]. The aim of the present study was to examine whether SkQ1 affects fast involution of the thymus in OXYS rats.

## Results

### Thymic involution in Wistar and OXYS rats at different ages

The thymus of OXYS rats, like that of Wistar rats, consists of two lobes of unequal size, which are tightly juxtaposed and separated by a thin layer of loose connective tissue. The anatomy of the thymus of OXYS and Wistar rats seem to be similar. However, microscopic studies of serial sections of OXYS rat thymus at 10 days of age revealed immature lobules which were remarkable for their lack of distinct differentiation between cortex and medulla as well as low cell density.

The absolute weight of the thymus in OXYS rats is significantly lower than in Wistar rats (Figure 1A-F). This is partially due to smaller body weight of OXYS rats. However, at age 2 and 3.5 months, not only absolute thymic weight but also thymic weight normalized to the body weight (thymic index) of OXYS rats was shown to be lower than that of Wistar rats (Figure 1B).

The total volume of the thymus significantly increased in both rat strains from age 10 days to age 2 months, reached a maximum, and then declined slightly between 2 and 3.5 months of age and significantly between 3.5 and 14 months of age. The total volume was always lower in OXYS rats, and the difference was statistically significant at age 10 days and 2 and 14 months (Figure 1C).

The thymic cortex volume in both strains of rats increased between 10 days and 2 months of age, reached a maximum, and then decreased (Figure 1D). At all time points, the thymic cortex volume was lower in OXYS rats, and statistically significant differences were observed at age 10 days and 2 and 14 months.

The volume of the medulla also increased in both strains of rats between 10 days and 2 months of age, reached a maximum at 3.5 months, and then declined (Figure 1E). The medulla volume was always lower in OXYS rats.

The ratio of cortex to medulla (C/M index) changed with age in a fashion similar in the two studied rat strains (not shown in figures).

The volume of connective tissue stroma of the thymus, the capsule, and septa changed as a function of age of the animals. It increased significantly between 10 days and 2 months of age, did not noticeably change between 2 and 3.5 months, and declined by 14 months in both rat strains. At all time points, the absolute value was lower in OXYS rats due to lower total thymic size (not shown).

One of the characteristic signs of age-related involution of the thymus is replacement of lymphoid tissue with adipose tissue. In the thymuses of 10-day-old Wistar and OXYS rats, adipose tissue was not detectable. At age 2 and 3.5 months, single adipocytes were observable in the capsule and septa. At age 14 months, adipose tissue occupied measurable part of the thymus. This volume was two-fold higher in Wistar rats compared to OXYS rats (2.8 ± 0.3 mm³ and 1.4 ± 0.2 mm³, respectively, *p* = 0.0033). Percent content of adipose tissue in the thymus of OXYS and Wistar rats was similar (12.6 ± 1.4 % vs. 13.5 ± 2.8 %, respectively).

**Figure 1A.**
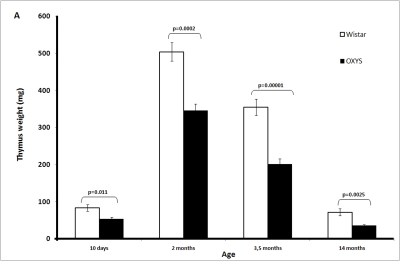
Age-related changes in the thymus of Wistar and OXYS rats. Absolute weight of the thymus (here and below, mean ± S.E.).

**Figure 1B.**
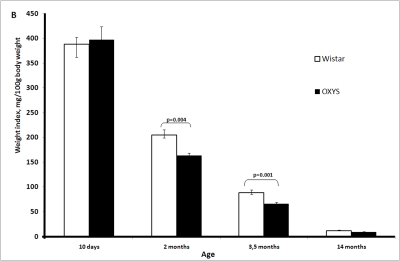
Age-related changes in the thymus of Wistar and OXYS rats. Thymic weight index.

**Figure 1C.**
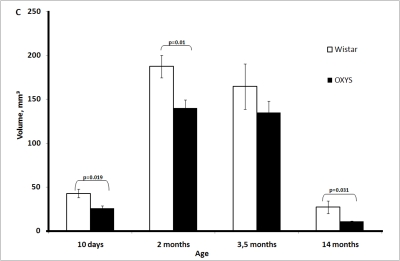
Age-related changes in the thymus of Wistar and OXYS rats. Total thymic volume.

**Figure 1D.**
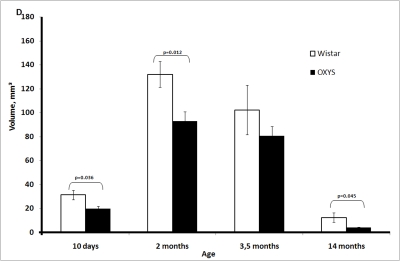
Age-related changes in the thymus of Wistar and OXYS rats. Volume of thymic cortex.

**Figure 1E.**
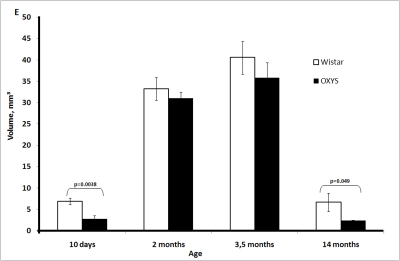
Age-related changes in the thymus of Wistar and OXYS rats. Volume of thymic medulla.

**Figure 1F.**
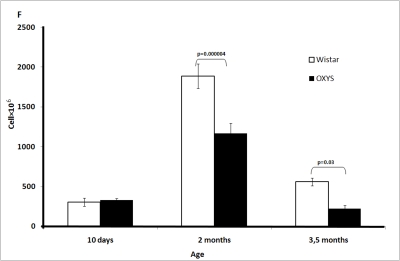
Age-related changes in the thymus of Wistar and OXYS rats. Total cell number (cellularity) of the thymus.

**Table 1.** Cellular content of the thymus in Wistar and OXYS rats at different ages (absolute numbers of various subpopulations of lymphocytes in the thymus, mean ± S.E. x10 ^6^).

Measurements	Age	Age group, ID (size)	Wistar	OXYS	Statistical significance of interstrain differences, *p*
CD3+	10 d.	1 (n=7)	2300±386	2308±17	not significant (>0.05)
	2 mo.	2 (n=7)	1550±131	859±92	0.0012
	3.5 mo.	3 (n=5)	405±35	168±43	0.0029
Comparison age groups, *p* value			1-2, =0.00031 2-3, =0.000123	1-2, =0.0001 2-3, =0.0001	
CD3+^hi^	10 d.	1 (n=7)	34.1±4.3	49.1±6.9	not significant
	2 mo.	2 (n=7)	227±9.4	162±17.2	0.0062
	3.5 mo.	3 (n=5)	90.1±13.6	38.9±6.8	0.0101
Comparison age groups, *p* value			1-2, <0.0001 2-3, <0.0001	1-2, =0.0001 2-3, =0.0002	
CD4+/ CD8+	10 d.	1 (n=7)	267±44.5	291±20.2	not significant
	2 mo.	2 (n=7)	1610±135.1	972±115.9	0.004
	3.5 mo.	3 (n=5)	481±44.2	192±36.4	0.0011
Comparison age groups, *p* value			1-2, <0.0001 2-3, <0.0001	1-2, =0.00013 2-3, =0.00013	
CD4+	10 d.	1 (n=7)	22.6±4.0	21.8±2.9	not significant
	2 mo.	2 (n=7)	173±18.9	136±9.8	not significant
	3.5 mo.	3 (n=5)	44.1±4.1	19.2±3.0	0.0014
Comparison age groups, *p* value			1-2, <0.0002 2-3, <0.0001	1-2, =0.00017 2-3, =0.00013	
CD8+	10 d.	1 (n=7)	6.1±1.2	6.4±0.8	not significant
	2 mo.	2 (n=7)	53.9±4.3	31.9±3.8	0.003
	3.5 mo.	3 (n=5)	23.1±3.1	7.4±1.2	0.002
Comparison age groups, *p* value			1-2, <0.00013 2-3, <0.0001	1-2, =0.00017 2-3, =0.00013	
CD4+/ CD8+	10 d.	1 (n=7)	3.8±0.1	3.4±0.3	not significant
	2 mo.	2 (n=7)	3.2±0.3	4.6±0.5	0.038
	3.5 mo.	3 (n=5)	1.9±0.1	2.6±0.2	0.002

At age 10 days and 2 and 3.5 months, the total number of cells (cellularity) in the thymus and immunological phenotype of thymocytes was determined using flow cytometry. Major subpopulations of thymocytes were detected by means of surface markers for CD3+, CD4+, and CD8+ cells. Two-way ANOVA analysis showed that the total cell number in the thymus changed with age. In both strains, it increased between 10 days and 2 months, reached a maximum, and then declined. No strain difference in total cell number was found on day 10. At the age of 2 and 3.5 months, this parameter was lower in OXYS than in Wistar rats (Figure 1F). Double-positive lymphocytes were the predominant cell type in the thymus at all time points in both strains. Their relative number was the highest in 10-day-old animals regardless of the strain. This measure tended to decrease with age (not shown).

The second most numerous subpopulation among thymocytes was mature CD4+ lymphocytes (Т-helper cells). Their number was not significantly different between the rat strains, but changed with age. The percentage of CD4+ cells was the lowest on day 10 in both rat strains, increased at age 2 months and decreased at age 3.5 months (Table 1).

The third most numerous subpopulation of thymocytes was CD8+ cells (cytotoxic T lymphocytes and suppressor T cells). Their dynamics were similar to that of CD4+ cells (Table 1). The relative number of all CD3+ thymocytes was dependent on both genotype and age of the animals. This parameter lowered with age in both rat strains, the lowering being stronger in the OXYS rats (Table 1). Analysis of subfractions of CD3+ cells showed that the content of CD3+^low+med^cells was lower in OXYS rats compared to Wistar rats (not shown). As to CD3+^hi^, it first increased and then decreased with age in both strains of rats (Table 1).

An important parameter of a population of single-positive thymocytes is the CD4+/CD8+ ratio. In the present study, it decreased with age in both strains (Table 1).

These results indicate that both OXYS and Wistar rats exhibit age-dependent signs of primary hypoplasia of the thymus, as evidenced by the lowering of average thymic weight and total volume, decrease in volumes of cortex and medulla (epithelial compartment of the thymus), as well as total numbers of thymic cells, and lowering of absolute numbers of major subpopulations of thymocytes. The direction of all these changes was similar in two strains. However, by the end of fast body growth and the beginning of age-related thymic invo-lution (age 2 to 3.5 months), lowering of the thymic cellularity developed more quickly in OXYS rats.

### Effect of mitochondria-targeted antioxidant SkQ1 on thymic characteristics of OXYS and Wistar rats

In the second part of the investigation, we studied the effect of SkQ1 on the morphological and functional state of the thymus of Wistar and OXYS rats. The animals received the drug with food at the dose of 250 nmol SkQ1/kg body per day weight from the age of 1.5 to 14 months.

Two-factorial dispersion analysis of weight measurements at age 3.5 months revealed that the drug treatment increases the absolute weight of the thymus (not shown) and thymic weight index (Figure 2A). In OXYS rats, the increase was stronger, being statistically significant. Similar relationships were revealed when the total thymic volume was measured (Figure 2B). The SkQ1 treatment also enhanced the volumes of structural components of the thymus - cortex, medulla, and connective tissue stroma. Again, the effect (a volume increase) was stronger in OXYS rats (Figure 2C-E).

The total number of cells in the thymus was affected by both drug treatment and genotype of the animals. The thymic cellularity in OXYS rats was much lower than in Wistar rats and was increased threefold by the SkQ1 treatment (Figure 2F).

Data on amounts of various cell fractions in the thymus of Wistar and OXYS rats treated and non-treated with SkQ1 are shown in Table 2. The table shows that percentage content of various subpopulations of thymo-cytes did not undergo significant changes as a result of the SkQ1 treatment. However, the analysis of absolute values revealed very strong positive effect of SkQ1.

Cell cycle analysis in thymocytes did not show significant differences between OXYS and Wistar rats. SkQ1 also had no effect on the percentage of cells in various phases of the cell cycle. The percentage of cells with fragmented DNA, characteristic of apoptosis (<G0), tended to be higher in OXYS rats and was lowered somewhat with the drug treatment, but these changes were not statistically significant (data not shown).

The number of thymocytes at the various stages of apoptosis was determined using parallel staining of the tissue from 3.5 months rats with annexin V, 7-amino-actinomycin D (7-ААD), or TUNEL followed by flow cytometry. No significant effects of strain or SkQ1 were revealed (not shown).

**Figure 2A.**
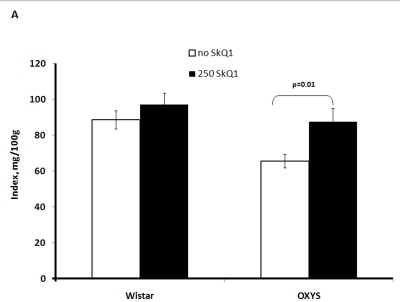
Effect of SkQ1 on thymic involution in Wistar and OXYS rats. Age of the animals, 3.5 months. SkQ1 was fed during the last 2 months.

**Figure 2B.**
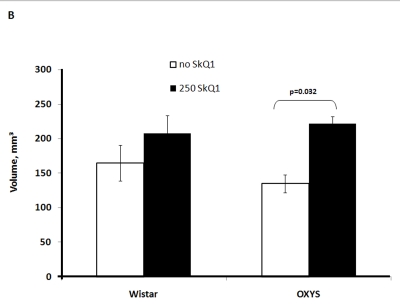
Effect of SkQ1 on thymic involution in Wistar and OXYS rats. Total thymic volume.

**Figure 2C.**
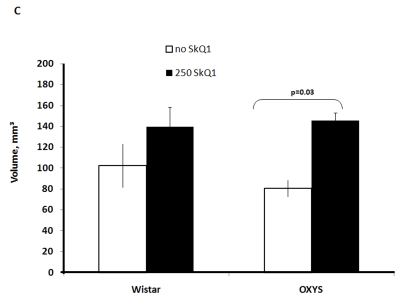
Effect of SkQ1 on thymic involution in Wistar and OXYS rats. Volume of thymic cortex.

**Figure 2D.**
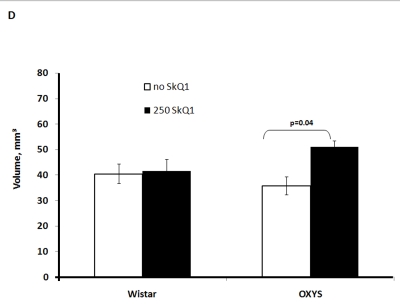
Effect of SkQ1 on thymic involution in Wistar and OXYS rats. Volume of thymic medulla.

**Figure 2E.**
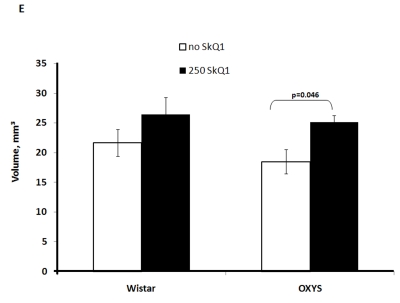
Effect of SkQ1 on thymic involution in Wistar and OXYS rats. Volume occupied by connective tissue stroma.

**Figure 2F.**
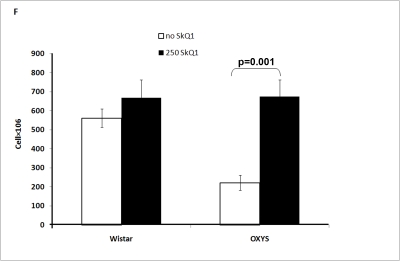
Effect of SkQ1 on thymic involution in Wistar and OXYS rats. Thymic cellularity.

In the last series of experiments, the effect of SkQ1 was studied on old (14 months) Wistar and OXYS rats. The data are summarized in Table 3. One can see pro-nounced SkQ1-induced increase in the thymic weight index, total volume of thymus, and volumes of its cortex and medulla. Volumes of thymic stroma and adipose tissue slightly or strongly increased, respectively. All the above changes with the exception of the adipose tissue volume were larger in Wistar than in OXYS rats (in con-trast to that observed in 3.5-month-old rats, see Figure 2A-F).

**Table 2.** Effect of SkQ1 on cellular content (mean ± S.E. X10 ^6 ^) of the thymus in Wistar and OXYS rats. Data for age 3.5 months with SkQ1 administration started at age 1.5 months.

Strain	Wistar		OXYS		Comparison of groups, *p* value
no SkQ1 (group 1)	SkQ1 (group 2)	no SkQ1 (group 3)	SkQ1 (group 4)
CD3+ (absolute)	405±35	526±73	168±43	488±66	1-3, =0.008 3-4, =0.002
CD3+^hi^ (absolute)	90.1±13.6	87.1±9.7	38.9±6.8	92.2±13.8	1-3, =0.018 3-4, =0.025
CD4+/CD8+(absolute)	481±44	588±85	191±36	582±72	1-3, =0.004 3-4, =0.001
CD4+(absolute)	44.1±4.1	47.9±5.6	19.2±3.0	49.3±9.5	1-3, =0.01 3-4, =0.017
CD8+(absolute)	23.1±3.1	21.6±2.9	7.4±1.2	27.4±5.8	1-3, =0.02 3-4, =0.008
CD4/CD8	1.9±0.1	2.3±0.2	2.7±0.2	1.8±0.1	1-2, =0.025 3-4, =0.017
CD3+ (%)	72.0±1.2	78.7±4.1	72.6±5.6	72.2±2.0	-
CD3+^low+med ^(%)	55.9±1.7	65.4±4.9	54.9±6.3	58.5±1.2	-
CD3+^hi ^(%)	16.1±2.1	13.3±1.2	17.6±1.0	13.7±1.1	-
CD4+/CD8+ (%)	85.5±1.4	87.6±0.6	85.6±1.1	86.6±1.1	-
CD4+ (%)	7.9±0.7	7.2±0.3	9.0±0.8	7.1±10.6	-
CD8+ (%)	3.8±0.4	3.9±0.3	3.7±0.2	3.5±0.2	-

**Table 3.** Effect of SkQ1 on the thymus (mean ± S.E.) in 14-month-old Wistar and OXYS rats.

Strain	Wistar		OXYS		Comparison of groups, *p* value
no SkQ1 (group 1) n=6	SkQ1 (group 2) n=9	no SkQ1 (group 3) n=7	SkQ1 (group 4) n=9
Thymic weight index (mg/100 g)	11.8±1.7	17.8±2.0	8.97±0.54	10.1±0.94	1-2, =0,008 2-4, =0,003
Total volume of thymus (mm^3 ^)	26.9±7.1	44.4±4.6	10.7±0.6	13.6±2.9	1-2, =0,007 1-3, =0,03 2-4, =0,0002
Volume of thymic cortex (mm^3 ^)	12.4±3.9	23.2±3.0	4.2±0.3	6.2±1.5	1-2, =0,005 2-4, =0,0003
Volume of thymic medulla (mm^3 ^)	6.7±2.1	12.7±1.4	2.4±0.1	3.6±0.9	1-2, =0,003 2-4, =0,0002
Volume of thymic stroma (mm^3 ^)	5.0±1.2	6.8±0.6	2.8±0.2	3.2±0.6	2-4, =0,002
Volume of adipose tissue (mm^3 ^)	2.8±0.4	1.7±0.3	1.4±0.2	0.7±0.2	1-2, =0,0035 1-3, =0,0011 2-4, =0,023

## Discussion

Age-dependent involution of the thymus is one of the best examples of programmed senescence [22-24]. It is clearly under control of genetic mechanisms responsible for ontogenesis, like, e.g., programmed involution of gills or tail in human embryo. The only difference between gills (or tail) involution and that of thymus consists in that they occur in the pre- and postnatal periods, respectively. In rats, absolute weight of thymus is maximal in 2-month-old animals and then strongly lowers with age down to 10-15% of maximal value at 14 months (Figure 1A). Such an involution is known to be species-specific, being much slower in long-lived than in short-lived organisms. This rule is also valid for strains of one and the same species. In our case, the thymic weight index of short-lived OXYS rats was equal to  the index  of long-lived  Wistar rats on day 10 but then decreased with age faster than that of Wistar rats (Figure 1B).

There are several pieces of evidence indicating that intramitochondrial ROS are necessary intermediates of the senescence program (for reviews, see [16,22,23]). This is why in our group a mitochondria-targeted rechargeable antioxidant SkQ1 was synthesized and tested as a potential geroprotector [16,25-28]. It was found that SkQ1 prolongs lifespan of different species: from podospora (a fungus) to mouse. In mouse, development of numerous traits of senescence were decelerated or stopped (in certain cases, even reversed) by very small amounts of SkQ1 [16,26]. In particular, it was found that SkQ1-treated mice kept in a non-sterile vivarium were resistant to various infections causing death of the non-treated control animals [16,26]. This effect was mainly responsible for a two-fold increase in the median lifespan of mice by SkQ1. This why we hypothesized that age-dependent decline of the immune system, being a part of the senescence program, is decelerated by SkQ1. Because thymic involution is one of the most important constituents of the decline of immune system with age, it was interesting to study the effect of SkQ1 upon this process.

Experiments were performed on a normal (Wistar) and a fast senescence-prone (OXYS) rat strains, the latter suffering from constant oxidative stress. In OXYS rats, involution of the thymus was shown to occur faster than in Wistar rats (Figure 1A-F, Table 1).

As to SkQ1, it decreased the rate of thymic involution in both studied strains, the drug action being more pronounced in OXYS rats at age 3.5 months and in Wistar rats at age 14 months. Small amounts of SkQ1 (250 nmol/kg per day with food) were found to strongly decrease such parameters of thymic involution as lowering of (i) absolute thymic weight, (ii) thymic weight index, (iii) total volume of thymus, (iv) volume of thymic cortex, (v) volume of thymic medulla, (vi) thymic cellularity, (vi) absolute amounts of the CD3+, CD4+, and CD8+ cells in the thymus, relative amount (percentage) of each cell type being unaffected (Figure 2A-F and Tables 2,3). These results are consistent with our suggestion that SkQ1 interferes with execution of a senescence program responsible, in particular, for age-dependent decline of the immune system [16].

## Methods

### Animals

In our experiments, we used males of OXYS rats at 10 days and 2, 3.5, and 14 months of age as well as age-matched male Wistar rats. The OXYS strain of rats was developed at the Institute of Cytology and Genetics, Siberian Division of the Russian Academy of Sciences, from Wistar stock by selection for their susceptibility to the cataractogenic effect of galactose [29]. To attain this goal, young Wistar rats were fed galactose-rich diets, and animals highly susceptible to the cataractogenic effect of those diets were selected for inbreeding. After five cycles of inbreeding, the subsequent generations of rats developed cataracts spontaneously, i.e. without galactose supplementation of the diet. This rat strain was called by the International Rat Genetic Nomenclature Committee as the OXYS rat strain [30]. At present, the strain of OXYS rats is maintained in the Breeding Experimental Animal Laboratory of the Institute of Cytology and Genetics.

The animals were housed in cages (45×35×35 cm) and kept under standard laboratory conditions (at 22 ± 1° C, 60% relative humidity, natural light), provided with a standard rodent diet (PK-120-1, Ltd. ‘Laboratorsnab', Russia) *ad libitum*. OXYS rats of all age groups had lower body weight compared to Wistar rats (21 ± 2 and 16 ± 1 g at day 10 (*p* < 0.007), 212 ± 5, and 247 ± 8 g at 2 months (*p* < 0.002), 307 ± 10 and 403 ± 11 g at 3.5 months (*p* < 0.001), and 404 ± 6 and 628 ± 29 g (*p* < 0.001) at 14 months of age, respectively.

The thymus was examined histopathologically and by morphometry. The cellular content of the thymus and immunologic phenotype of thymic cells were measured by flow cytometry.

To determine the effects of long-term dietary SkQ1 supplementation on the thymus of the experimental animals, we started with 1.5-month-old males of OXYS and Wistar rat strains. The rats were randomly assigned to one of two groups: a control diet and diet supplemented with SkQ1 (250 nmol/kg of body mass per day). The latter group received SkQ1 up to the age of 14 months.

All the experiments on rats were carried out in line with Animal Care Regulations of Institute of Cytology and Genetics, Novosibirsk.

### Histology and morphometric characteristics

The thymus was weighed to estimate the weight index (mg thymus/100 g body weight), fixed in Telesnitsky fixative at 4°C, dehydrated in alcohols of increasing grade, cleared in xylene, and embedded in Paraplast Plus. Serial frontal sections were cut at 5 μm and stained with hematoxylin and eosin. Each 60^th^ section was examined by light microscopy. Morphometry of structural components of the thymus was carried out by stereologic techniques and the absolute total thymic volume and volumes of structural components were calculated.

### Flow cytometry

Thymic cellularity and immunologic phenotype. The thymus was placed in RPMI-1640 medium with 2% fetal calf serum and 1% sodium azide. Single-cell suspension of thymocytes was prepared and the total number of thymocytes was determined. A 100-μl aliquot of the cell suspension was incubated with a fluorochrome-conjugated antibody at room temperature for 15 min. The following monoclonal antibodies (all from BD Pharmingen, San Diego, Calif., USA) were used for staining: FITC-conjugated anti-CD3 (Cat. No. 559975), PE-conjugated anti-CD4 (Cat. No. 551397), and FITC-conjugated anti-CD8a (Cat. No. 554856). After staining, the cells were washed twice with PBS containing 0.1% sodium azide and fixed in PBS with 1% formaldehyde. The samples were analyzed by flow cytometry on the FACS Calibur instrument (Becton Dickinson, USA) using Cell Quest computer software.

Analysis of apoptosis. The frequency of apoptosis was estimated by flow cytometry using an annexin V-PE apoptosis detection Kit I (BD Pharmingen, Cat. No. 559763) and an APO-BrdU Kit (BD Pharmingen, Cat. No. 556405) according to the manufacturer's instructions.

Cell cycle analysis *. *A sample of 10^5 ^cells was fixed with 70% ethanol, washed with PBS, incubated for 20 min with 0.5 ml propidium iodide solution (10 μg/ml in PBS) plus RNase (5 mg/ml) in the dark at room temperature. Samples were analyzed by flow cytometry on the FACS Calibur instrument using Cell Quest computer software. The percentages of cells within G0/G1, S, G2/M phases and <G0 were determined.

Statistical analysis. The data were analyzed using ANOVA with the statistical package Statistica 6.0. The independent variables were the genotype for comparison between OXYS and Wistar rats and the age for comparison between 10 days and 2, 3.5, and 14 months of age. Two-way ANOVA (age×genotype) was used to evaluate the differences between Wistar and OXYS rats across different ages. To test the effect of the SkQ1, the strain and antioxidant were chosen as independent variables. A Newman−Keuls *post hoc* test was applied to significant main effects. One-way ANOVA was used for individual group comparisons.
